# Early-childhood BMI trajectories: evidence from a prospective, nationally representative British cohort study

**DOI:** 10.1038/nutd.2016.6

**Published:** 2016-03-07

**Authors:** B Stuart, L Panico

**Affiliations:** 1 Senior Research Fellow, Primary Care and Population Sciences, Faculty of Medicine, University of Southampton, Aldermoor Health Centre, Aldermoor Close, Southampton, UK; 2 Institut National d'Etudes Démographiques (Ined), Paris, France

## Abstract

**Background and Objectives:**

By age 5, 20% of British children are classed as overweight or obese, suggesting that early childhood is crucial for lifelong body mass index (BMI) trajectories. In this paper, we identify latent trajectories of early-childhood BMI from ages 3 to 11 years. Given the current context of growing socio-economic inequalities in childhood and adult overweight and obesity, we examine the socio-economic characteristics and mechanisms during pregnancy and infancy which underscore these trajectories.

**Subject and Methods:**

We use a nationally representative, prospective cohort study of 9699 children born in 2000–2002, living in the United Kingdom shortly after birth, with complete information on height and weight (measured by an interviewer) at ages 3, 5, 7 and 11. Trajectories of BMI are calculated using latent growth mixture models. Multinomial models characterize these trajectories by their socio-economic profiles and mechanisms during pregnancy and infancy.

**Results:**

Four trajectories were identified: two separate trajectories where BMI remains within a normal range (85% of the sample), an overweight trajectory (14.4%), and an obese trajectory (3.1%). No ‘declining BMI' or late-onset groups were found. The obese group is already distinct from the other trajectories by age 3. The overweight group diverges from the normal groups around age 5. Strong socio-economic inequalities emerged; for the obese group, part of this disadvantage is mediated through early mechanisms such as pregnancy smoke and not initiating breastfeeding.

**Conclusions:**

This study provides strong evidence for the idea that childhood BMI trajectories develop early, especially for children who will follow an obese trajectory. Strong socio-economic patterns in these trajectories suggest that the observed trend in growing inequalities may be rooted in early life. Mediating mechanisms for the obese appear to be in the pregnancy and infant period, further research should explore mechanisms occurring around age 5 when the overweight trajectory diverges.

## Introduction

Childhood body mass index (BMI) predicts adulthood obesity^1, 2^ and other long-term health outcomes, such as high cholesterol or asthma.^3, 4, 5, 6, 7^ Yet, the dynamic processes that drive the development of childhood overweight and obesity are not completely understood, especially in early childhood, both within the context of academic research and for clinical and public health practice. This knowledge could enhance our understanding of the development of BMI trajectories, and can guide clinical and public health practice by highlighting early-life mechanisms occurring before BMI trajectories are ‘set'. The early years appear to be critical for the development of lifelong BMI trajectories: by 5 years of age, over 20% of British children are already classed as overweight or obese,^8^ and studies suggest that the vast majority of excess weight appears to be already gained by age 5, especially for girls.^9^

In England, while rates of overweight and obesity among school-age children appear to have stabilized in recent years,^10^ children from lower socio-economic strata have not benefited from this trend,^11^ suggesting a widening of inequalities. Such inequalities appear to be already in place from early childhood, but little research has attempted to identify differences in BMI trajectories across socio-economic groups. Better identifying the interplay between early-life socio-economic factors and BMI trajectories in childhood may help understand growing gap in overweight and obesity prevalence across socio-economic groups.

Exploring BMI trajectories from early childhood requires large sample sizes and fine measures of weight and height: to our knowledge; most studies in this field use dichotomous measures of obesity and/or overweight, problematic as it can create misclassification bias, especially at young ages. Other studies that do use continuous measures of BMI do not adjust for age and sex, which may risk not accounting for important variation in growth; this is again especially problematic in early childhood. Only a few studies have examined trajectories in BMI from early childhood, adjusting for both age and sex;^12, 13, 14^ however, the resulting trajectories have not been replicated. These studies use regional populations, and, combined with their relatively small sample sizes, may produce results that are not generalizable and therefore not replicated. The Millennium Cohort Study allows us to address these issues.

The first objective of this study is to use group-based trajectory modelling to examine BMI trajectories in early childhood in a large, prospective, and nationally representative cohort study of British children born in 2000–2002, the Millennium Cohort Study. Within the context of growing socio-economic inequalities in childhood overweight and obesity, our second and third objectives are to examine the socio-demographic profiles of these trajectories and to explore the mechanisms in pregnancy and infancy, which may underlie the relationship between socio-economic background and BMI trajectories in early childhood, before these trajectories are set.

## Data and methods

### The Millennium Cohort Study

The Millennium Cohort Study is a nationally representative birth cohort study with a sample drawn from infants born from September 2000 to January 2002 and living in the United Kingdom shortly after birth. It follows 18 553 households that agreed to participate in the initial survey, an overall response rate of 68%. Households were identified through the Department of Work and Pensions Child Benefit system and selected on the basis of where the family was resident shortly after birth. Uptake of Child Benefit is almost universal (98%).

The sample has a probability design and is clustered at the electoral ward level, with disadvantaged residential areas and areas with a high proportion of ethnic minority population being over represented.^15^ For classification purposes, we use data from the second, third, fourth and fifth sweep of interviews, carried out through home visits when the cohort member was aged ~3, 5, 7 and 11 years, respectively. Data were collected through home interviews with the main carer, usually the mother. The overall sample size for sweep 2 was 15 307, 15 246 at sweep 3, 14 043 at sweep 4 and 13 287 at sweep 5. This analysis is based on 9699 cases with complete information on the child's weight and height at ages 3, 5, 7 and 11 years.

### Variable description

At ~3, 5, 7 and 11 years of age, trained interviewers recorded the child's weight using Tanita scales, and measured their height. This was carried out during the home visit. These measurements are used to calculate the child's BMI. BMI was calculated as weight in kilograms divided by height in metres squared. Owing to the interest in developmental trajectories over the full age range, only cases with complete information at all sweeps on these two key variables (weight and height) were retained (*n*=9699). BMI is modelled continuously in latent analyses, but in descriptive statistics and to characterize the BMI trajectories, we use overweight and obese thresholds based on the cutoffs determined for each age and gender by the International Obesity Taskforce.^16^ Although BMI is often used to assess whether an individual's body weight deviates from what is anticipated for a person's height, it cannot account for adiposity or other factors such as muscularity. Therefore, although cutoffs for overweight and obesity based on BMI are widely used, these thresholds are valid only as statistical categories and not to accurately diagnose individual level overweight or obesity.

A number of markers for socio-economic background were considered. A forward selection exercise was carried out to reduce the number of variables included in the model. Forward selection is a data-driven model-building approach in which variables are added to the model one at a time and tested for inclusion in the model. The most significant of these variables are retained in the model, as long as its *P*-value is below a pre-set level. It is customary to set this value above the conventional 0.05 level because of the exploratory nature of this method. The exercise begins with the variable that appears to be most significant in initial analyses, and continue adding variables until none of remaining variables is ‘significant' when added to the model. After this excercise, we retained in the regression models three socio-economic variables, which were significant at the level *P*=0.20: parental income, parental education, and a persistent poverty indicator. This allowed us to keep the model relatively simple while maximising its predictive power. The first two variables are based on the first interview with the main carer, carried out when the child was ~9-month old. Annual parental income is modelled as a log-transformed measure; and parental education is operationalised by the highest educational qualification for either resident parents (no educational qualifications; only overseas qualification; equivalent qualifications to an National Vocational Qualification (NVQ)1, NVQ2, NVQ3, NVQ4 and NVQ5. As an indicator, an NVQ5 is equivalent to a graduate degree; an NVQ3 is equivalent to two A-levels, a high-school qualification. The NVQs is a system of competence-based education and training that aims to recognise and record individual levels of competence. It covers all educational levels, from primary school to university. The framework indicates the ‘equivalence' of both vocational and academic qualifications, and reflects the level of skills acquired. There is no further detail for those who did not have a British educational qualification, they are therefore classed separately. A persistent poverty indicator, which captures the frequency the household was classed as poor over the 5 waves of data collection (from 9 months to 11 years of age), was calculated using data from those sweeps. Households were classed as poor if their equivalised income was 60% below the mean income for that wave. Equivalised income takes into account household composition and was calculated using the Organisation for Economic Co-operation and Development (OECD)'s modified equivalence scale.

To identify factors in pregnancy and early childhood that may be related to future BMI, the following variables, measured at the 9-month interview (sweep 1) as reported by the main respondent, were retained in the final models: any smoking by the mother during pregnancy; whether the child was never breastfeed, low birthweight (<2500 grams at birth) and high birthweight (birthweight of or over 4500 grams). All these variables are coded as binary (yes/no) variables.

We also retain two control variables in the model, the child's sex and their ethnicity, the latter was classed as a binary variable indicating whether the child was White or from any other ethnicity.

### Statistical analysis

First, using group-based trajectory modelling (a semi-parametric mixture model), we determine whether there are groups of individuals who follow similar longitudinal patterns of BMI over childhood. These models allow us to identify distinct trajectories for two or more latent groups and the estimated prevalence of these groups within the study population. Parameters are estimated using maximum likelihood.

To fit the models, we used the Stata traj plug-in, with BMI modelled as censored normal. Survey weights were applied to account for the clustered nature of the survey design. Models were fitted iteratively starting with one latent trajectory (which assumes that all children follow the same trajectory) and fitting up to six latent trajectory groups. The models were first fitted assuming a cubic relationship and dropping down to quadratic or linear for any non-significant polynomial terms.^17^ Model fit was assessed by comparing BIC values using the log Bayes factor,^18^ the significance tests of the model coefficients and conceptual usefulness, avoiding trajectories that represented <1% of the study population. Individuals were allocated to the trajectory for which they have the highest membership probability. As BMI patterns differ for boys and girls, the analysis was carried out for boys and girls separately, as well as for the whole cohort.

Second, descriptive analyses and multinomial logistic regressions were carried out in Stata 13 to characterize the BMI trajectories. Multinomial regression was used to determine the association between the socio-economic variables and mechanisms during infancy and pregnancy and BMI trajectories. Three sequential models were tested: the first model only controlled for sex and ethnicity, the second introduced socio-economic variables, and the final model added potential pregnancy and infancy mechanisms. All analyses presented include the appropriate survey weights.

## Results

### Description of the study sample

Table 1 reports the cross-sectional proportion of children with BMIs above the appropriate age- and sex-specific overweight and obese cutoffs at 3, 5, 7 and 11 years of age in our analytical sample (*n*=9699). This shows that by age 11, ~20% of our sample was overweight and 5% was obese. Following the concept of adiposity rebound (a normal pattern of growth, that, after a decline of BMI-for-age from ~1 to 5–6 years, shows an increase in adiposity through to adolescence,^19^ the proportion of children overweight or obese follows a U-shaped curve across the study period, with peaks at 3 and 11 years, and the lowest proportions at 7 years of age. From age 5, girls were more likely than boys to be classed as overweight or obese.

### Trajectories

**Figure 1:**
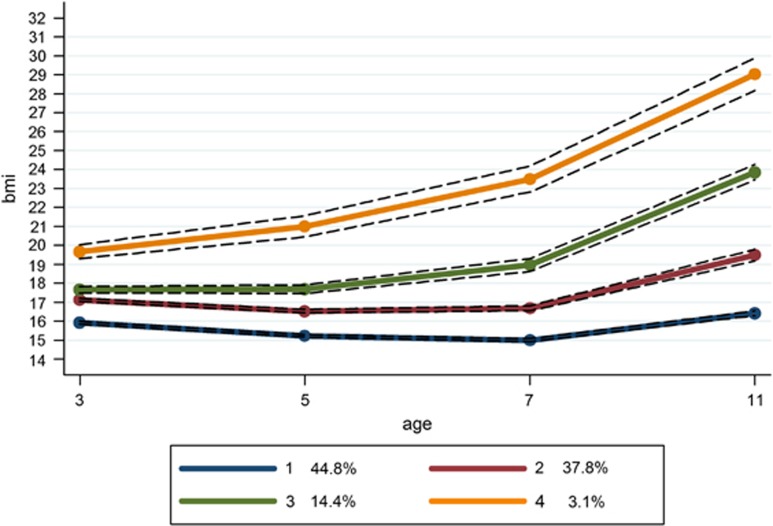
BMI trajectories for the entire sample, from ages 3 to 11, with 95% confidence intervals.

**Figure 2:**
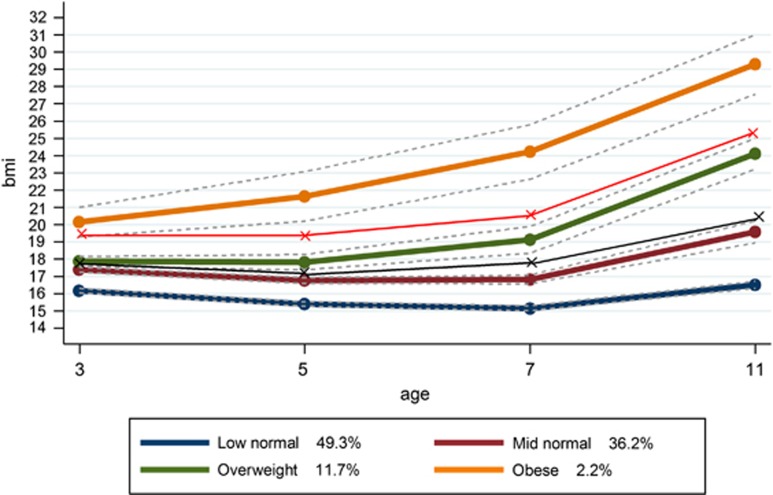
BMI trajectories for boys, from ages 3 to 11, with age- and sex-specific cutoffs for overweight and obesity.

**Figure 3:**
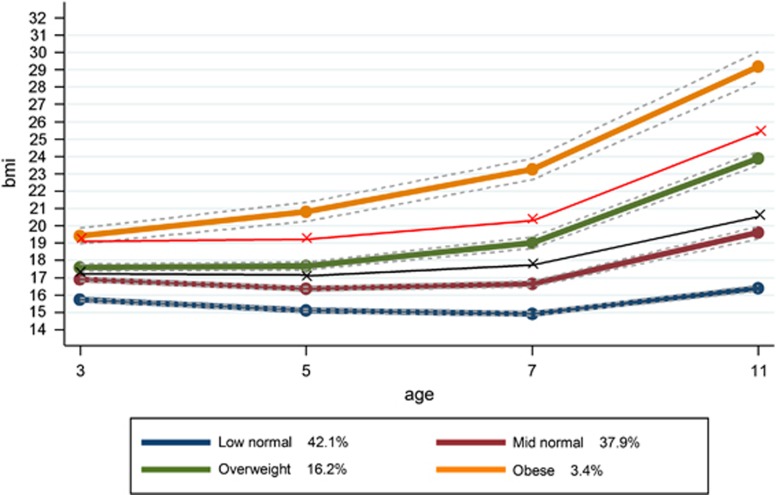
BMI trajectories for girls, from ages 3 to 11, with age- and sex-specific cutoffs for overweight and obesity.

Using latent techniques, four distinct trajectories were identified for all children (Figure 1) and for both boys and girls separately (Figures 2 and 3, respectively). When these trajectories are compared with the recommended cut-offs for overweight and obese, the following groups can be identified. Group 1 represents children who were at low to normal BMI throughout the study period, retaining an average BMI of ~16. This was the largest group, at 44.8% of the study population. Group 2 was the second largest at 37.8% of the population. This group started at a somewhat higher average BMI at the age of 3 than Group 1 and was only just below the threshold for ‘overweight' at this point. Their average BMI remained consistently below the ‘overweight' cutoff throughout the study period. We refer to Group 1 as ‘low-normal' and Group 2 as ‘mid-normal'. Taken together, this suggests that 82.6% of the children in the study were on ‘normal' BMI trajectories that stayed below overweight cutoff marks.

Group 3 appears to be similar to the mid-normal group at 3 years of age but their trajectory then develops in a different direction. Instead of slightly decreasing through 5–7 years as a normal pattern of growth would predict, their BMI continues to increase, keeping them above the ‘overweight' cutoff throughout the observed period, but always below the ‘obese' cutoff. This ‘overweight' trajectory represents 14.4% of the population. A further 3.1% of children were classed in an ‘obese' trajectory. Their average BMI was already above the cutoff for obesity at the 3 years of age and their trajectory shows an increasing BMI, continuing which remains above the obese cutoffs at all ages. Although the patterns for boys and girls are very similar, there is a slightly higher prevalence of overweight and obesity for girls. The model did not identify a trajectory of decreasing BMI, nor a trajectory suggesting a change from overweight or obesity to a normal BMI. Supplementary information shows the mean BMI for each group, by sex and at each age, as well as the sample size for each group by sex.

### Predictors of trajectories

As expected, Table 2 shows that the overweight and obese groups have a similarly higher proportion of girls than boys (43% boys), whereas the low-normal and mid-normal groups have about an equal division of boys and girls. Children from a White ethnic background were most represented in the mid-normal group, and least likely to be represented in the obese group.

Table 2 also shows the distribution of the socio-economic variables and potential early mechanisms by BMI trajectories. It shows that the two ‘normal' BMI groups are more likely to be represented in the top income quintiles, whereas the obese group appears to have the lower proportions of children in the richest income group. The overweight and the obese group have a similar proportion of their sample in the bottom income quintile. However, the low-normal group is slightly more likely to be represented in the poorest quintile than the mid-normal group.

The patterns for parental education confirm these following differences: the low-normal group appears to have the highest proportion of parents in the top educational qualifications groups, whereas the obese the smallest proportion. The obese group has the highest proportion of parents classed as having no educational qualifications or low levels of educations, whereas the two normal groups had the smallest proportion in these two groups at 3.7%. The overweight group is in an intermediate position between the two normal groups and the obese group at both the top and bottom of the education qualifications distribution.

Looking longitudinally, less than half of the obese group never experienced poverty at the five data points collected, and 7% of this group was classed as poor at every data point, marking it out as the most disadvantaged group in this domain. The low-normal group was the most likely to be classed as never poor and the mid-normal the least likely to be classed as always poor. As above, the overweight group is an intermediate position between the two normal groups and the obese group.

Table 2 also reports the prevalence of the potential intermediate mechanisms during pregnancy and infancy. The low-normal had the highest proportion of low birthweight babies, the mid-normal the smallest proportion, whereas the obese trajectory had the highest proportion of high birthweight babies. The low-normal group was least likely to report maternal smoking during the pregnancy and in infancy, whereas the obese group was the most likely. For smoking, a gradient was evident, with the mid-normal group reporting the second lowest rates followed by the overweight group. This gradient was also found for breastfeeding initiation, where the low-normal group had the highest rates and the obese the lowest, with the mid-normal and overweight in between.

To disentangle the relative importance of the variables discussed above, multinomial models were employed to predict the risk of belonging to a BMI trajectory relative to belonging to the mid-normal trajectory (always used as the reference group). Results are presented in Table 3. Model 1 includes the child's sex and ethnicity, and confirms the bivariate findings that boys are slightly less likely to belong to the overweight and obese groups relative to the mid-normal group. It also confirms that White children are more likely to be represented in all groups compared with the mid-normal group, this effect is particularly strong for the obese compared with the mid-normal group.

Model 2 adds the three socio-economic variables. We find no important differences in socio-economic background between the low- and mid-normal groups, except for a small but significant effect of being less likely to belong to the low-normal group compared with the mid-normal group if the household was sometimes (but not always) poor rather than never poor during the study period. The overweight group did not appear to have different educational profiles, but as income increased, membership to the overweight group compared with the mid-normal group decreased, suggesting that children from low-income families are more likely to be classed in the overweight than in the mid-normal trajectory. Inversely, comparing the obese versus mid-normal group suggests that although household income did not predict membership between these two classes, education did, with lower parental education predicting a higher chance of belonging to the obese rather than the mid-normal group.

Model 3 adds potential mechanisms relating to infancy and pregnancy. This model suggests that low birthweight strongly predicts membership to the low-normal rather than the mid-normal groups, and a smaller effect of smoking at 9 months also predicts belonging to the low-normal rather than the mid-normal group. However, adding these variables to the model did not change the risk ratios for the socio-economic variables and controls, suggesting that low birthweight and parental smoke in infancy did not mediate the lower propensity of White children and children who were sometimes poor to belong to low-normal rather than the mid-normal group. For the comparison of the overweight versus mid-normal group, none of the mechanisms added to the model appear to be significant, and the income coefficient was not changed between model 2 and model 3. On the other hand, for the obese versus mid-normal group, being born at a high birthweight, not being breastfed and maternal smoke during the pregnancy both predicted a higher risk of belonging to the obese rather than the mid-normal group, and these variables appear to significantly decrease the education coefficients seen in model 2, suggesting that the higher risk of children of low educated parents to belong to the obese rather than the mid-normal trajectory might at least partially transit through these two mechanisms. However, they did not attenuate the sex or ethnicity coefficients, suggesting that other mechanisms are at play there.

## Discussion

In this work, we use a growth mixture-modelling approach to identify distinct trajectories of BMI between 3 and 11 years of age, estimated using a large, nationally representative cohort of children living in the United Kingdom. Four trajectories were identified: two trajectories, a low-normal and a mid-normal trajectory, where BMI remains within a normal range (comprising 85% of our sample), an overweight trajectory (14.4%), and an obese trajectory (3.1%). A recent review of similar studies using group-based trajectory modelling or growth mixture modelling determined that four population subgroups were most common, with stable high and low categories, and rising and declining categories.^12^

Notable findings include: (1) an early-onset, chronically obese group can be identified, this group is already well above the average BMI by 3 years of age, (2) trajectories for boys and girls are remarkably similar, (3) differently from other studies,^12, 20^ no ‘declining' group was found, nor did we find early- and late-onset groups, adding evidence to the idea that tracking (the concept of persistence or relative stability of excess weight over time, which appears to drive overweight trajectories in adolescents and adults^21^) occurs from a very early age and that once overweight or obese trajectories are established they are difficult to change; however (5) the mid-normal and the overweight groups appear very similar at 3 years, and only diverge by 5 years of age. This suggests that there may still be some movement between normal and overweight BMI before age 5, whereas the obese trajectory appears to be set from age 3. The differences we find from other studies, notably the fact that no declining trajectory is found, could be due to the fact that our sample is a national sample, which includes a relatively disadvantaged population, which is often missing by more specific or community-based samples.

Characterizing these BMI trajectories by their socio-economic profiles showed that socio-economic inequalities are already evident in this young age group. We show that the overweight and obese groups are more disadvantaged than the normal groups, but in slightly different ways: the overweight group seems to be mostly characterized by low parental incomes, whereas the obese group by low parental education. The fact that childhood overweight and obesity is linked to socio-economic status is not new,^11^ although here we show that socio-economic status does not uniformly impact BMI trajectories, and different indicators of disadvantage (for example, parental incomes or education) seem to capture different trajectories.

Factors in infancy and pregnancy did not seem to mediate the relationship between lower incomes and increased membership to the overweight compared with the mid-normal group, which resonates with our finding that the overweight and mid-normal trajectories do not appear to substantially diverge until age 3–5 years. However, high birthweight, maternal smoking during the pregnancy and not being breastfed did mediate some of the educational gradient between the obese and the mid-normal group. This again resonates with our result that the obese trajectory is already distinct by age 3, suggesting that very early-life factors may be at play for the development of this trajectory.

Using longitudinal data, and characterizing BMI trajectories by their socio-economic profiles and by potential underlying mechanisms is useful in that it sheds light on groups where intervention would be most helpful. Our results suggest that the preschool period is ripe for attempting to modify these trajectories, whereas interventions in primary or secondary schools being perhaps less useful to prevent obesity.

The results of this study should be interpreted taking account of important limitations. First, owing to missing data, the final model was estimated using only about half of the original cohort, introducing a potential source of bias. As most longitudinal studies, loss to follow-up in the Millennium Cohort Study is greater in children from more socially deprived backgrounds, it is therefore likely that children excluded from our analyses because of missing data had higher rates of overweight and obesity than those included in our analyses. We therefore may underestimate the relationship between markers of socio-economic background and the risk of following an overweight or obese trajectory; that is, socio-economic inequalities in the development of overweight and obesity may actually be even more important than what shown here. Second, our analytical technique shares the limitations of other clustering techniques. Even though researchers use a number of statistical fit criteria as a guide, the problem of determining the number of classes has not been completely resolved. Furthermore, it should also be noted that latent trajectories are not directly observed clusters but groups constructed on the basis of the pattern of responses over a fixed number of observation periods. These methods therefore do not predict the development of BMI in an individual, nor produce overall population prevalences, instead trajectories are derived by assigning each child a probability of membership based on their overall longitudinal symptom history. Finally, although we have accurate measures of children's height and weight, we caution that while a high BMI is usually synonymous with overweight or obesity, this is not always the case. BMI does not measure adiposity and cannot account for other factors such as muscularity. Therefore, some children with a high BMI may not be overweight or obese, and thresholds based on BMI are not valid tools to accurately diagnose individual-level overweight or obesity.

Although there were some limitations to this study, there were also numerous strengths. Our data's prospective, longitudinal, population-based design representative at a national level and spanning a large portion of early and mid-childhood, is a major strength. This study adds to a literature often using small, regional samples of children, by being able to use a large and representative sample, allowing us to include nearly 10 000 children in our analytical sample, to use an age- and sex-adjusted continuous measure of BMI rather than dichotomous measures of overweight or obesity, which is especially important in early childhood to avoid misclassification, and to carry out analyses separately for boys and girls. The rich Millennium Cohort Study data allowed us a large enough sample size to explore a number of important socio-economic variables and potential mechanisms. Height and weight were directly measured by trained interviewers; this is important as parental reports tend to result in overestimates overweight and obesity, especially at younger ages.^22^

Early-life factors may be crucial in setting up lifelong BMI trajectories, which are in turn associated to lifelong health and mortality. Our results suggest tracking may be an important concept even from early childhood, especially for obesity. Overweight and obesity trajectories both seem to be linked to more disadvantaged family backgrounds, but not in the same way and working through different mechanisms. Our findings suggest that, for obesity, mechanisms in pregnancy and infancy are crucial, whereas for the overweight trajectory, further research should focus on potential mechanisms in the 3–5 age group. These results suggest that the early years may be critical for tackling growing socio-economic inequalities in childhood overweight.

## Figures and Tables

**Table 1:** Proportion of children overweight, obese, mean weight and mean BMI at 3, 5, 7 and 11 years (weighted analyses)

	*Boys*				*Girls*			
	Age 3	Age 5	Age 7	Age 11	Age 3	Age 5	Age 7	Age 11
% overweight	17.4%	13.3%	11.2%	18.3%	17.4%	16.5%	15.2%	21.8%
% obese	4.4%	4.2%	4.3%	5.0%	4.7%	4.8%	5.2%	5.7%
Mean weight (kg)	15.7	20.2	25.5	40.4	15.0	19.7	25.2	41.9
Mean BMI	16.9	16.3	16.4	18.8	16.6	16.2	16.5	19.3
Sample size (unweighted)	4.619	4.621	4.620	4.620	4.752	4.754	4.753	4.749

Abbreviation: BMI, body mass index.

**Table 2:** Socio-economic variables and exposure variables, by latent trajectories (weighted percentages)

	*Low normal*	*Mid normal*	*Overweight*	*Obese*
*Socio-economic variables*				
Parental income, 9 months				
Poorest quintile	10.2	9.2	14.6	14.9
2	16.3	17.2	20.0	21.6
3	15.4	17.0	19.5	17.7
4	25.4	27.2	22.0	26.4
Richest quintile	32.7	29.4	23.9	19.4
*P*-value	<0.0001			
Highest parental education, 9 months				
None of these	3.9	3.7	5.7	7.7
Overseas quals only	1.1	1.1	1.4	3.1
NVQ 1	3.9	3.7	4.5	9.4
NVQ 2	20.4	22.5	26.5	29.0
NVQ 3	15.8	15.9	17.5	16.1
NVQ 4	45.3	44.5	38.0	31.5
NVQ 5	9.5	8.6	6.3	3.3
*P*-value	<0.0001			
Persistent poverty indicator				
Never poor	65.0	62.7	54.5	47.3
Sometimes poor	30.1	33.6	39.7	45.8
Always poor	5.0	3.8	5.8	7.0
*P*-value	<0.0001			

*Exposures*				
Low birthweight	8.9	5.2	6.4	6.8
High birthweight	1.1	2.1	2.3	3.6
Smoked during pregnancy	16.8	18.2	22.4	33.2
Either parent smokes, w1	36.2	39.7	46.5	49.8
Breastfeeding initiated	78.3	77.0	72.1	66.9

*Controls*				
Gender				
Male	50.3	50.9	43.3	43.8
*P*-value	0.0003			
Ethnicity				
non-White	10.0	8.2	12.1	17.0
*P*-value	<0.0001			

Abbreviation: NVQ, National Vocational Qualification.

**Table 3:** Multinomial logistic regression of socio-economic and early-life factors by latent trajectories (weighted relative risk ratios)

	*Model 1* *RRR*	*Model 2* *RRR*	*Model 3* *RRR*
Mid normal		Reference class	

*Low normal*			
Not breastfed			0.96
Parental smoke, 9 months			1.14*
Smoke during pregnancy			1.00
Low birthweight			1.93***
High birthweight			0.45***
Log weekly income in pounds, 9 months		1.02	1.01
No educational quals		1.05	1.05
Overseased quals only		1.08	1.08
NVQ 1		1.04	1.05
NVQ 2		0.89	0.90
NVQ 4		0.98	0.95
NVQ 5		1.01	1.00
Sometimes poor		0.87*	0.87*
Always poor		1.21	1.26
Gender (child is male)	0.98	0.96	0.97
Ethnicity (child is white)	0.82*	0.80*	0.83

*Overweight*			
Not breastfed			1.15
Parental smoke, 9 months			0.84
Smoke during pregnancy			1.01
Low birthweight			1.21
High birthweight			1.08
Log weekly income in pounds, 9 months		0.83**	0.84**
No educational quals		1.26	1.22
Overseased quals only		1.03	1.00
NVQ 1		0.98	0.93
NVQ 2		0.99	0.95
NVQ 4		0.82	0.84
NVQ 5		0.75	0.78
Sometimes poor		0.99	0.96
Always poor		1.01	0.95
Gender (child is male)	0.74***	0.75***	0.74***
Ethnicity (child is white)	0.65***	0.72**	0.68**

*Obese*			
Not breastfed			1.33*
Parental smoke, 9 months			1.04
Smoke during pregnancy			1.96***
Low birthweight			1.17
High birthweight			2.16*
Log weekly income in pounds, 9 months		1.05	1.09
No educational quals		1.85*	1.65
Overseased quals only		2.41*	1.91
NVQ 1		2.59**	1.94*
NVQ 2		1.29	1.24
NVQ 4		0.78	0.87
NVQ 5		0.40*	0.45*
Sometimes poor		1.27	1.19
Always poor		1.09	0.83
Gender (child is male)	0.76*	0.73*	0.73*
Ethnicity (child is white)	0.44***	0.46***	0.36***

Abbreviation: NVQ, National Vocational Qualification. ***P<0.001, **P<0.01, *P<0.05.

## Supplementary Material

Supplementary Informations
