# Supplementary material for: Early-childhood BMI trajectories: evidence from a prospective, nationally representative British cohort study
Source: Nutr Diabetes. 2016 Mar 7;6(3):e198–. doi: 10.1038/nutd.2016.6 (PMC4817077; doi:10.1038/nutd.2016.6)
Supplement: Supplementary Informations [file nutd20166x1.docx]

| **Supplementary information: Mean BMI and sample size by latent trajectories** | | | | |
| --- | --- | --- | --- | --- |
|  | **Low normal** | **Mid normal** | **Overweight** | **Obese** |
| **Boys** |  |  |  |  |
| **Mean BMI, age 3** | 16.1 | 17.3 | 17.8 | 19.9 |
| **Mean BMI, age 5** | 15.3 | 16.7 | 17.7 | 21.2 |
| **Mean BMI, age 7** | 15.1 | 16.7 | 19.0 | 23.8 |
| **Mean BMI, age 11** | 16.3 | 19.4 | 23.9 | 28.9 |
| **% (unweighted sample size)** | 49.3% (2,082) | 36.2% (1,793) | 11.7% (597) | 2.2% (150) |
| **Girls** |  |  |  |  |
| **Mean BMI, age 3** | 15.7 | 16.7 | 17.6 | 19.4 |
| **Mean BMI, age 5** | 15.1 | 16.4 | 17.7 | 20.8 |
| **Mean BMI, age 7** | 14.9 | 16.7 | 19.1 | 23.3 |
| **Mean BMI, age 11** | 16.4 | 19.7 | 24.0 | 29.2 |
| **% (unweighted sample size)** | 42.1% (2,037) | 37.9% (1,737) | 16.2% (798) | 3.1% (182) |
